# Open Reduction and Intrafragmentary Compression Fixation with External Fixator (the Ichi-Fixator) Treatment of Distal Phalangeal Nonunion

**DOI:** 10.1155/2020/8878002

**Published:** 2020-08-20

**Authors:** Satoshi Ichihara, Yasuhiro Yamamoto, Akira Hara, Masao Suzuki, Yuichiro Maruyama

**Affiliations:** ^1^Hand Surgery Center, Juntendo University Urayasu Hospital, Chiba, Japan; ^2^Department of Orthopedic Surgery, Juntendo University, Tokyo, Japan; ^3^Department of Orthopedic Surgery, Juntendo University Urayasu Hospital, Chiba, Japan

## Abstract

The nonunion of distal phalangeal communized fracture is relatively rare in hand fractures. However, if it occurred, the surgical treatment is quite difficult because of small piece of fragmentations. We developed a new fixation method that involves the insertion of two wires and external wire compression fixation using a metal clamp. The aim of this technique was to increase the compression force between fragments and rigidity of conventional percutaneous Kirschner wire fixation. Here, we present a patient with the nonunion of distal phalangeal communized fracture who was satisfactorily treated with open reduction and percutaneous interfragmentary compression fixation with a linking external wire fixator (the Ichi-Fixator system). Such a treatment that enables compression fixation for communized distal phalangeal fracture of nonunion will clearly boost bone healing. Linked external wire-type compression fixator (the Ichi-Fixator system) enables enhanced security of fixation and facilitates the bone healing.

## 1. Introduction

Distal phalangeal fractures can be managed conservatively with good outcomes if diagnosed early. However, if the initial reduction is imperfect with total displacement between fragments such as communized fracture, then it may be necessary to use headless screw or Kirschner wire (K-wire) for fixation after closed or open reduction. Moreover, if conservative treatment failed, fracture site was occupied by soft tissue and fibrous connective tissues and finally become nonunion. Treatment of nonunion will need to firmly fix at fracture site after sufficient refreshment for increasing the bone union rate. The treatment options for nonunion of distal phalangeal fracture include conservative management with use of ultrasound [1], bone graft with K-wire fixation [2], bone peg transplantation [3], and open reduction with internal fixation with a headless compression screw [4]. Various treatment options have been described, but no clear consensus on the management of symptomatic nonunion of distal phalangeal fractures has yet emerged [5]. Furthermore, when left untreated, or if the reduction is incomplete, such injuries can lead to symptomatic nonunion, manifesting as chronic pain and/or instability. To overcome these complications, we developed a new linking wire type of external fixator (the Ichi-Fixator system: Neo-medical, Saitama, Japan) that involves the insertion of two wires and external wire compressional fixation with special adjustable metal clamp fixation using small two inside screws [6]. The aim of using this technique is to increase the compression force to the nonunion site and rigidity of conventional percutaneous K-wire fixation. Here, we present a patient with nonunion of distal phalangeal fracture who was satisfactorily treated with open reduction and percutaneous fixation with the Ichi-Fixator system.

## 2. Case Report

**Figure 1:**
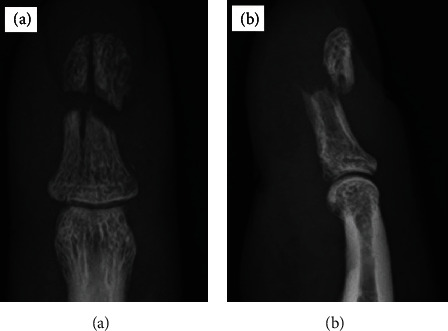
Radiographs showing nonunion of the distal phalanx of the right middle finger 10 weeks after injury: (a) AP view; (a) lateral view.

A 45-year-old man presented with severe pain following conservative plaster treatment for 10 weeks at another hospital. He was referred our hospital for severe pain and chronic instability of the tip of right middle finger during manual work. A diagnosis of nonunion of the 3^rd^ distal phalangeal fracture was made based on radiographs and computed tomography scans. Anteroposterior, lateral, of the right middle finger showed dorsal displacement of the distal phalangeal fracture, with communized fracture (Figure 1). Finally, we decided to perform external fixation using the Ichi-Fixator system with linked-wire external fixator under locoregional anaesthesia in the operating room. Before the operation, the quick disabilities of the arm, shoulder, and hand questionnaire (QDASH) score was 84.09 and the visual analogue scale (VAS) pain score was 8/10.

## 3. Surgical Technique

**Figure 2:**
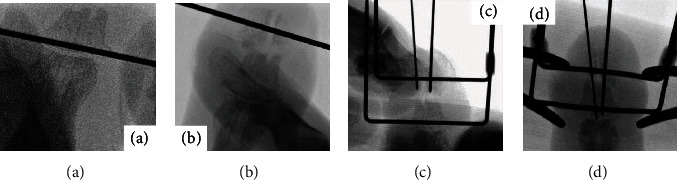
Intraoperative X-ray. (a) A 1.2 mm fixator pin was inserted from the base of the distal phalangeal bone transversally. (b) A 1.2-mm fixator pin was inserted from the distal one-third of the distal phalangeal bone transversally. (c) The parallel ends of the two pins were inserted. (d) Compression of fracture with Kocher.

**Figure 3:**
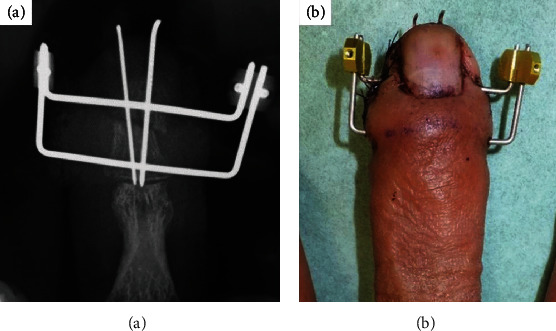
Postoperative radiographic (a) and clinical views (b).

**Figure 4:**
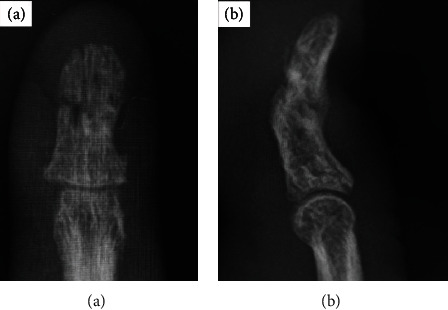
Union of distal phalanx nonunion after 10 weeks: (a) AP view; (b) lateral view.

**Figure 5:**
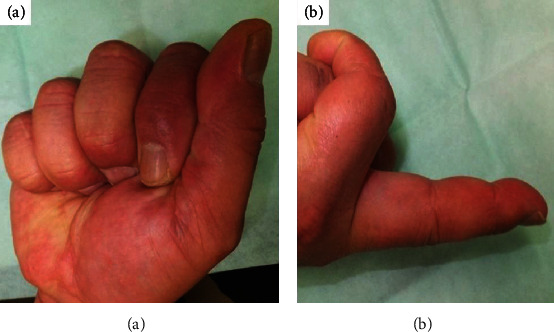
The range of motion of the operated finger after 10 weeks: (a) total flexion; (b) extension of the right middle finger.

Surgery was performed by a highly experienced surgeon. Curved joystick incision was performed at the lateral of distal phalangeal bone, and sufficient refreshment between the fracture sites with use of curettage was done [7]. After open reduction under fluoroscopic inspection, a 0.7 and 0.9 mm Kirschner wire was inserted from the tip of the middle finger along the longitudinal direction to the DIP joint; then, a 1.2 mm fixator pin was inserted from the base of the distal phalangeal bone transversally (Figure 2(a)). Another 1.2 mm fixator pin was inserted from the distal one-third of the distal phalangeal bone transversally (Figure 2(b)). The external four ends of the two pins were bent so that they became parallel. The parallel ends of the two pins were then inserted in a metal clamp from the same sides (Figure 2(c)). After fluoroscopic assessment, two parallel pins were inserted, compression was performed with the use of Kocher (Figure 2(d)), the fracture site is close to each other, and the ends of the two pins were fixed firmly together using two clampers (Figure 3). Before definitive fixation, each side of the compression force adjusts with use of small screws inside of the fixator. The patient was allowed to start range-of-motion exercises immediately after the operation. The pins were removed in an outpatient setting when union of the fracture was verified radiologically. In the current case, the linked-wire external fixator was in place for 8 weeks. At the most recent follow-up, the nonunion was united (Figure 4), the QDASH score was 2.27, and the VAS score was 1/10. The grip strength and total active motion were 86.7% and 94.7%, respectively, compared with the contralateral side (Figure 5, Supplementary Material). The patient achieved almost normal grip strength and showed no pain and no instability at the nonunion site, then returned to all his previous activities without discomfort.

## 4. Discussion

The distal phalanx nonunion is rare, and specific guidelines for their treatment do not exist [5]. In the previous literature, there are few reports regarding surgical treatment for nonunion of distal phalanx. Voche et al. treated 13 patients by using open reduction and K-wire or small cancellous screw. As postoperative complication, stiffness of the distal interphalangeal joint, sensory loss of the fingertip, necessity of an arthrodesis, resection of the distal fracture fragment, and amputation of the distal phalanx occurred [8]. Chim et al. described 14 patients treated with open reduction and screw fixation. Although they reported good function results, it was necessary to remove screw for screw protrusion, screw senility, and fingertip tenderness [3]. Kim et al. reported to perform bone grafts using peg bone in 13 patients. There were no severe complications and all united [4]. These reports were mostly nonunion of simple transverse fractures. In our case of longitudinal communized fracture, the fracture may be extended by using screws or peg bone. Whereas conventional K-wire fixation usually uses two wires to achieve cross pinning, the Ichi-Fixator system facilitates reliable two- or three-dimensional fixation. Such reliable fixation guards against secondary displacement after primary operation. Moreover, in contrast to conventional pinning, patients can start range-of-motion exercises immediately after the operation. In a previous study, locked wire systems were found to better resist loosening [9, 10]. One of the advantages of the Ichi-Fixator system is easy to compress between two fragments. Especially, in this case, because of the quite narrow space between two bone fragments, it is difficult to insert metal clamp in this area. The most appealing point of the characteristic of Ichi-Fixator system is the adjustable functions. After the insertion of two pins, free directional bending of the two pins and external compressional fixation were done using a metal clamp. Two pins enable to temporary fix together with a special adjustable metal clamp with two small screws. In this case, we gradually fix with each side of the screw under fluoroscopic inspection. Another advantage of the Ichi-Fixator system is patient comfort after the operation. Patients do not need to guard against or fear K-wires loosening or becoming detached. It may be that the linkage of K-wires will allow the omission of all additional external splintage. This should minimize joint stiffness and the consequent need for hand therapy. Such a treatment that improves comfort after the operation and may allow an immediate return to work will clearly boost patient satisfaction.

## 5. Conclusions

We experienced the open reduction and intrafragmentary compression fixation with an external fixator (the Ichi-Fixator system) treatment of distal phalangeal nonunion. This system enables to increase the compression force at the nonunion site compared with conventional percutaneous K-wire fixation.

## Data Availability

This is my "Data Availability" statement as follows. The Figures data used to support the findings of this study are included within the article. The Supplementary data used to support the findings of this study are included within the supplementary information file(s).

## Supplementary Materials

Supplementary materials Video data: range of motion after surgery at 2, 14, and 70 days.
